# Prevalence and Determinants of Low Serum Vitamin D Among Women Attending Infertility Clinics in Japan: A Real‐World Multicenter Cross‐Sectional Study

**DOI:** 10.1002/rmb2.70032

**Published:** 2026-02-22

**Authors:** Kuniaki Ota, Toshihiro Habara, Nobuo Koyama, Yoshiharu Nakaoka, Takeshi Kuramoto, Mamoru Seki, Hiroaki Yoshida, Takashi Kuno, Motoo Nabeta, Takahito Miyake, Masayuki Kinutani, the JISART Vitamin D Study Group

**Affiliations:** ^1^ Department of Obstetrics and Gynecology Kawasaki Medical School Okayama Japan; ^2^ Fukushima Medical Center for Children and Women Fukushima Medical University Fukushima Japan; ^3^ Okayama Couple's Clinic Okayama Japan; ^4^ ART Clinic for Female Kumamoto Japan; ^5^ IVF Namba Clinic Osaka Japan; ^6^ Kuramoto Women's Clinic Fukuoka Japan; ^7^ Sekiel Ladies Clinic Gunma Japan; ^8^ Sendai ART Clinic Miyagi Japan; ^9^ Tsukasa Clinic Sakai‐Higashi Osaka Japan; ^10^ Tsubaki Women's Clinic Ehime Japan; ^11^ Miyake Clinic Oayama Japan; ^12^ Kinutani Women's Clinic Hiroshima Japan

**Keywords:** 25‐hydroxyvitamin D, assisted reproductive techniques, infertility, Japan, prevalence, vitamin D

## Abstract

**Purpose:**

To estimate the prevalence and correlates of low serum 25‐hydroxyvitamin D [25(OH)D] among women attending infertility clinics in Japan.

**Methods:**

We conducted a multicenter cross‐sectional study of 17 261 women with measured 25(OH)D. Deficiency and insufficiency were defined as < 20 and < 30 ng/mL. Monthly/seasonal variation was assessed, and multivariable logistic regression evaluated correlates of deficiency. In a complete‐case subset with vitamin D supplement and multivitamin information, 25(OH)D levels and 3‐category status (deficient/insufficient/sufficient) were compared between users and non‐users. The association between diminished ovarian reserve (DOR) and vitamin D status categories was assessed using the chi‐square test.

**Results:**

Median 25(OH)D was 16.4 ng/mL; 66.8% were deficient and 89.0% insufficient. Levels varied by month and season (*p* < 0.001). Higher BMI was associated with higher odds of deficiency, whereas older age was associated with lower odds. Supplement users had higher 25(OH)D and a more favorable 3‐category distribution (*p* < 0.001); notably, 94.0% of supplement‐free women were insufficient (< 30 ng/mL). Vitamin D status categories were not significantly associated with DOR (*p* = 0.238).

**Conclusions:**

Low 25(OH)D is highly prevalent in infertility care in Japan with marked seasonal variation and adiposity‐related vulnerability. Supplement use was associated with higher 25(OH)D but did not explain the overall high burden.

## 1 Introduction

Vitamin D is a pleiotropic secosteroid hormone involved not only in calcium homeostasis but also in immune and endocrine regulation. Growing evidence has expanded interest in vitamin D beyond skeletal health, and the widespread observation that serum 25‐hydroxyvitamin D [25(OH)D] levels correlate with diverse health outcomes has led to increased testing and supplementation worldwide, even as recent guidance underscores uncertainty regarding causality and optimal targets for many non‐skeletal endpoints [1].

In reproductive medicine, vitamin D has attracted attention because vitamin D receptors and metabolizing enzymes are expressed in reproductive tissues, and vitamin D may influence folliculogenesis, steroidogenesis, endometrial receptivity, and immune tolerance. Recent reviews have highlighted these molecular and endocrine pathways as plausible links between vitamin D status and female reproductive health [2]. Clinical studies have continued to explore whether low [25(OH)D] is associated with infertility‐related conditions and ART outcomes. A 2023 systematic review of IVF studies summarized heterogeneous findings, with some evidence suggesting better outcomes among women with higher vitamin D status, while emphasizing substantial methodological variability across cohorts [3]. More recently, a 2024 meta‐analysis reported an association between vitamin D levels and clinical pregnancy and live birth rates, though heterogeneity and threshold effects remained notable [4]. In women with PCOS, a 2023 meta‐analysis suggested that vitamin D supplementation may improve ovulation and pregnancy rates and favorably modulate androgen and gonadotropin profiles, yet the authors also highlighted the need for higher‐quality trials [5].

From an epidemiologic perspective, defining the burden of low vitamin D in infertility populations is essential for interpreting interventional evidence and designing targeted preventive strategies. The commonly used thresholds defining deficiency (< 20 ng/mL) and insufficiency (21–29 ng/mL) originate from established endocrine guidance and remain widely applied in reproductive studies [6]. However, reported prevalence varies substantially by geography and latitude, as well as by lifestyle and body composition. Large pooled analyses in the general population have demonstrated marked global and regional heterogeneity in vitamin D deficiency, with women and younger adults often identified as vulnerable groups, and meta‐analytic evidence consistently links adiposity with a higher prevalence of low 25(OH)D levels [7, 8]. In addition, vitamin D–containing supplement use can shift the distribution of 25(OH)D and may differ by age or clinic practice patterns, complicating interpretation of clinic‐based prevalence estimates if supplementation is not considered. Accordingly, robust real‐world data that describe both vitamin D status and supplementation patterns are necessary to contextualize mixed evidence on fertility and ART outcomes and to inform pragmatic counseling or supplementation strategies.

In Japan, where latitude spans a wide range and seasonal ultraviolet exposure varies markedly, robust nationwide, real‐world data describing vitamin D status in infertility clinics remain limited. Our previous large single‐center study in East Japan demonstrated clear monthly/seasonal variation in [25(OH)D] among reproductive‐aged women with infertility, with the lowest levels in winter, while suggesting no straightforward linear relationship with ovarian reserve markers or Th1/Th2 ratio [9]. These findings underscore the need for broader multicenter evaluation to clarify how common low vitamin D status is across Japanese infertility settings, which clinical factors are most strongly associated with deficiency, and how vitamin D–containing supplement use relates to the observed [25(OH)D] distribution in routine practice.

Therefore, this multicenter cross‐sectional study aimed to estimate the prevalence of low serum [25(OH)D] levels among women attending infertility clinics in Japan and to identify associated factors, including age, BMI, infertility diagnosis, hormonal profiles, and the month/season of measurement, with consideration of inter‐clinic and geographic variation. We additionally examined vitamin D status according to vitamin D–containing supplement use (vitamin D supplements and/or multivitamin preparations containing vitamin D) in a complete‐case subset to aid interpretation of the observed [25(OH)D] distribution in real‐world infertility practice.

## 2 Material and Method

### 2.1 Study Design and Setting

This retrospective, multicenter, real‐world, database‐based cross‐sectional observational study analyzed routinely collected real‐world clinical data from women attending participating infertility clinics in Japan. The study aimed to estimate the prevalence of low serum 25(OH)D levels and to identify clinical factors associated with low vitamin D status in real‐world infertility practice.

### 2.2 Participants

Women who visited the participating infertility clinics and had serum 25(OH)D measured during the study period were eligible. We additionally excluded physiologically implausible 25(OH)D values (e.g., extreme values suggestive of data entry error) by treating them as missing prior to analysis. Non‐numeric or non‐interpretable 25(OH)D entries (e.g., values recorded with inequality symbols) were treated as missing. For analyses requiring the timing of measurement, only records with a recorded month/date of 25(OH)D assessment were included.

### 2.3 Data Source and Variables

Clinical and questionnaire‐based variables were extracted from a standardized data sheet used across clinics. Dichotomous variables were coded as 1 (present/yes) and 0 (absent/no). The dataset included demographic characteristics (age), anthropometrics (body mass index [BMI]), infertility‐related diagnoses (e.g., ovulatory, tubal, uterine, and male factors), lifestyle factors (e.g., smoking, alcohol use, exercise), supplementation status (e.g., folic acid, multivitamins, vitamin D), and selected hormonal/biochemical parameters (e.g., AMH, FSH, LH, estradiol, progesterone, prolactin, testosterone, TSH, free T4), depending on measurement availability. The month of serum 25(OH)D assessment was used to evaluate monthly and seasonal variation in this real‐world cohort.

To account for geographic variability, clinic‐level latitude was assigned based on clinic location and merged with the clinical dataset where applicable.

### 2.4 Definitions of Low Vitamin D Status

The primary outcome was vitamin D deficiency defined as serum 25(OH)D < 20 ng/mL. A secondary outcome of vitamin D insufficiency was defined as 25(OH)D < 30 ng/mL, according to widely used Endocrine Society guidance and prior reproductive studies [1, 6]. For analyses requiring mutually exclusive categorization, vitamin D status was classified as deficient (< 20 ng/mL), insufficient (20–< 30 ng/mL), or sufficient (≥ 30 ng/mL).

### 2.5 Data Cleaning and Handling of Implausible Values

To minimize the influence of apparent data entry errors, implausible values were treated as missing for descriptive and regression analyses based on pre‐specified criteria for age and BMI. Extreme vitamin D values considered incompatible with physiological ranges were handled in the same manner. Records with non‐numeric or non‐interpretable laboratory entries were also treated as missing. All cleaning rules were applied prior to generating descriptive summaries and regression models.

### 2.6 Missing Data

Missing values were expected due to the real‐world nature of the dataset. Descriptive statistics were calculated using available data for each variable. Primary multivariable analyses were performed using a complete‐case approach for selected covariates. Variables with substantial missingness, particularly lifestyle and supplementation items, were evaluated in sensitivity analyses when feasible. Because supplementation variables were not uniformly available across clinics and had higher missingness, they were not included in the primary multivariable models and were examined in separate analyses.

### 2.7 Seasonal Classification

Seasons were defined a priori based on the month of 25(OH)D measurement as follows: winter (December–February), spring (March–May), summer (June–August), and autumn (September–November). If the exact date was available, the calendar month was derived from that date; otherwise, the recorded month field was used.

### 2.8 Statistical Analysis

Continuous variables were summarized as mean ± standard deviation (SD) or median (interquartile range [IQR]), as appropriate. Categorical variables were summarized as numbers and percentages.

Monthly and seasonal distributions of 25(OH)D were visualized using box‐and‐whisker plots. For graphical clarity, outliers beyond 1.5 × IQR are not displayed in the box plots but were retained in all statistical analyses. Group differences across months or seasons were assessed using the Kruskal–Wallis test, followed by Dunn's post hoc test with multiplicity adjustment.

The prevalence of vitamin D deficiency (< 20 ng/mL) and insufficiency (< 30 ng/mL) was calculated overall and stratified by clinic and season. When presenting deficiency and insufficiency as cutoffs (< 20 and < 30 ng/mL), we note that these proportions are not additive because insufficiency includes deficiency. Using mutually exclusive categories (deficient/insufficient/sufficient), group differences were assessed using the chi‐square test.

Multivariable logistic regression was performed to identify factors associated with vitamin D deficiency. Covariates considered clinically relevant a priori included age, BMI, infertility diagnosis categories, and clinic; season of measurement was additionally included in a secondary model. Where applicable, clinic latitude was added as a geographic proxy for ambient ultraviolet exposure. Adjusted odds ratios (aORs) with 95% confidence intervals (CIs) were reported. Primary multivariable models did not include supplementation variables due to missingness and between‐clinic availability. Hormonal/biochemical parameters were not included in the primary multivariable models unless explicitly specified, given variable availability across clinics.

In an additional analysis, serum [25(OH)D] status was compared according to vitamin D–containing supplement use. Women were classified as supplement users if they reported current intake of either a vitamin D supplement or a multivitamin preparation containing vitamin D; all others were categorized as non‐users. Among women with complete information on both vitamin D and multivitamin supplementation and a valid [25(OH)D] measurement, median [25(OH)D] levels were compared between supplement users and non‐users using the Mann–Whitney *U* test, and the mutually exclusive 3‐category vitamin D status distribution (deficient/insufficient/sufficient) was compared using the chi‐square test. Additionally, the association between vitamin D–containing supplement use and vitamin D deficiency was evaluated as a sensitivity analysis using multivariable logistic regression restricted to records with complete supplementation data.

Two‐sided *p* values < 0.05 were considered statistically significant. Analyses were performed using EZR (Saitama Medical Center, Jichi Medical University) and/or R (R Foundation for Statistical Computing, Vienna, Austria) [10].

### 2.9 Ethics Statement

This study was conducted in accordance with the Declaration of Helsinki. The study protocol was approved by the institutional review board of Japanese Institution for Standardizing Assisted Reproductive Technology (approval No. 2024‐24). Because this was a retrospective analysis of de‐identified data, the requirement for informed consent was waived according to institutional policy.

## 3 Result

A total of 17 306 records were available from participating infertility clinics. After data cleaning and excluding records without a valid serum 25(OH)D value, 17 261 women were included in the primary analyses. Specifically, non‐numeric 25(OH)D entries and physiologically implausible extreme values were treated as missing and excluded from primary analyses.

### 3.1 Patient Characteristics

Baseline characteristics of the study population and data completeness for key variables are summarized in Table 1. The mean age was 34.9 ± 4.9 years (available data *n* = 14 216) and the mean BMI was 21.7 ± 3.4 kg/m^2^ (*n* = 15 290). The median serum 25(OH)D level was 16.4 ng/mL (IQR, 12.3–22.3). Infertility‐related diagnoses and lifestyle/supplementation variables were collected using a standardized data sheet; however, the availability of some variables varied across clinics, reflecting real‐world practice. Accordingly, descriptive statistics are reported based on available data for each item. For lifestyle and supplementation variables, data were available only for a subset of women (e.g., regular exercise, *n* = 263); therefore, these items are presented descriptively and should be interpreted with caution because of low response rates.

**TABLE 1:** Baseline characteristics of women attending infertility clinics with available serum 25‐hydroxyvitamin D measurements.

Characteristic	Overall (*N* = 17 261)
Age, years	34.9 ± 4.9 (*n* = 14 216)
Gravidity, *n*	0.8 ± 1.1 (*n* = 10 867)
Parity, *n*	0.3 ± 0.6 (*n* = 10 865)
BMI, kg/m^2^	21.7 ± 3.4 (*n* = 15 290)
Infertility duration, months	24 (12–42) (*n* = 8042)
Serum 25(OH)D, ng/mL	16.4 (12.3–22.3) (*n* = 17 261)
History of cesarean delivery	581 (8.1%) (*n* = 7177)
*Lifestyle factors*	
Alcohol use	24 (13.3%) (*n* = 180)
Smoking	162 (3.4%) (*n* = 4806)
Regular exercise	3 (1.1%) (*n* = 263)
*Supplement use*	
Folic acid supplementation	456 (9.9%) (*n* = 4629)
Multivitamin supplementation	65 (2.3%) (*n* = 2841)
Vitamin D supplementation	1314 (7.6%) (*n* = 17 256)
*Comorbidities*	
Hypertension	271 (5.5%) (*n* = 4951)
Diabetes mellitus	23 (0.5%) (*n* = 4951)
Dyslipidemia	41 (1.2%) (*n* = 3398)
*Infertility diagnoses*	
Endometriosis	1485 (8.6%) (*n* = 17 260)
Corpus luteum insufficiency	100 (0.6%) (*n* = 17 261)
Diminished ovarian reserve	2538 (14.7%) (*n* = 17 261)
Polycystic ovary syndrome (PCOS)	2338 (13.5%) (*n* = 17 261)
Ovulatory factor	2316 (13.4%) (*n* = 17 261)
Tubal factor	2344 (13.6%) (*n* = 17 261)
Uterine factor	1739 (10.1%) (*n* = 17 261)
Male factor	5833 (33.8%) (*n* = 17 261)
Unexplained	6149 (35.6%) (*n* = 17 261)

*Note:* Data are presented as mean ± SD or median (IQR), and *n* (%), as appropriate.

### 3.2 Prevalence of Low Vitamin D Status

The overall prevalence of vitamin D deficiency, defined as 25(OH)D < 20 ng/mL, was 66.8% (11 524/17 261). When using the secondary threshold of 25(OH)D < 30 ng/mL, 89.0% (15 360/17 261) of women were classified as having vitamin D insufficiency. Overall and season‐stratified prevalence estimates are shown in Table 2, providing a nationwide real‐world snapshot of vitamin D status among women seeking infertility care in Japan. When vitamin D status was presented as mutually exclusive categories (deficient < 20, insufficient 20 to < 30, sufficient ≥ 30 ng/mL), the distribution differed significantly across seasons (chi‐square test, *p* < 0.001; Table 2).

**TABLE 2:** Distribution of serum 25‐hydroxyvitamin D categories, stratified by season and vitamin D supplement use.

	*N*	Deficient < 20 ng/mL, *n* (%)	Insufficient 20 to < 30 ng/mL, *n* (%)	Sufficient ≥ 30 ng/mL, *n* (%)	*P*
Overall	17261	11 524 (66.8%)	3836 (22.2%)	1901 (11.0%)	
Winter	3751	2597 (69.2%)	742 (19.8%)	412 (11.0%)	< 0.001
Spring	4028	2736 (67.9%)	818 (20.3%)	474 (11.8%)	
Summer	3838	2467 (64.3%)	925 (24.1%)	446 (11.6%)	
Autumn	3835	2324 (60.6%)	1025 (26.7%)	486 (12.7%)	
*Vitamin D supplement use*					
No	15942	10 775 (67.6%)	3401 (21.3%)	1766 (11.1%)	< 0.001
Yes	1314	748 (56.9%)	434 (33.0%)	132 (10.0%)	

*Note:* Deficient < 20 ng/mL; Insufficient 20 to < 30 ng/mL; Sufficient ≥ 30 ng/mL. *P* values are from chi‐square tests comparing the 3‐category distribution across seasons (Winter–Autumn) and across vitamin D supplement use (Yes vs. No); shown once per block. Seasonal analyses include only participants with available measurement month.

### 3.3 Monthly and Seasonal Variation

**FIGURE 1:**
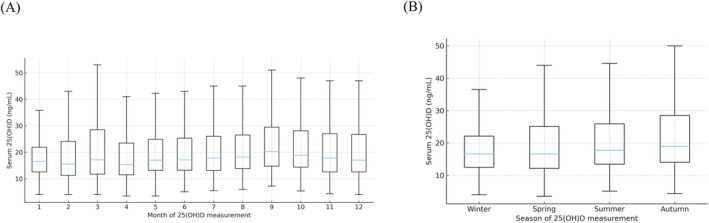
Monthly and seasonal variation in serum 25‐hydroxyvitamin D [25(OH)D] among women attending infertility clinics in Japan. (A) Box‐and‐whisker plots of serum 25(OH)D concentrations by month of assessment. (B) Box‐and‐whisker plots of serum 25(OH)D concentrations by season of assessment (winter: December–February; spring: March–May; summer: June–August; autumn: September–November). In each box plot, the center line indicates the median, the box indicates the interquartile range (IQR), and whiskers extend to 1.5 × IQR. For visual clarity, outliers beyond 1.5 × IQR are not displayed but were retained in all statistical analyses. Overall group differences were assessed using the Kruskal–Wallis test. Pairwise post hoc comparisons were performed using Dunn's test with multiplicity adjustment; statistically significant differences are indicated on the graphs (adjusted *p* values as shown).

Among women with recorded month of 25(OH)D assessment, serum 25(OH)D distributions differed significantly across months (Kruskal–Wallis test, *p* < 0.001; Figure 1A). The highest median values were observed in September (median 20.35 ng/mL).

Seasonal differences were likewise significant (Kruskal–Wallis test, *p* < 0.001). Median 25(OH)D levels were 16.6 ng/mL in winter (IQR, 12.5–22.1), 16.6 ng/mL in spring (12.1–25.1), 17.7 ng/mL in summer (13.4–25.9), and 18.9 ng/mL in autumn (14.0–28.5). Vitamin D deficiency (< 20 ng/mL) was most frequent in winter (69.2%) and least frequent in autumn (60.6%; Table 2, Figure 1B). Post hoc pairwise comparisons are provided in the figure legends.

### 3.4 Vitamin D Status According to Supplementation

**FIGURE 2:**
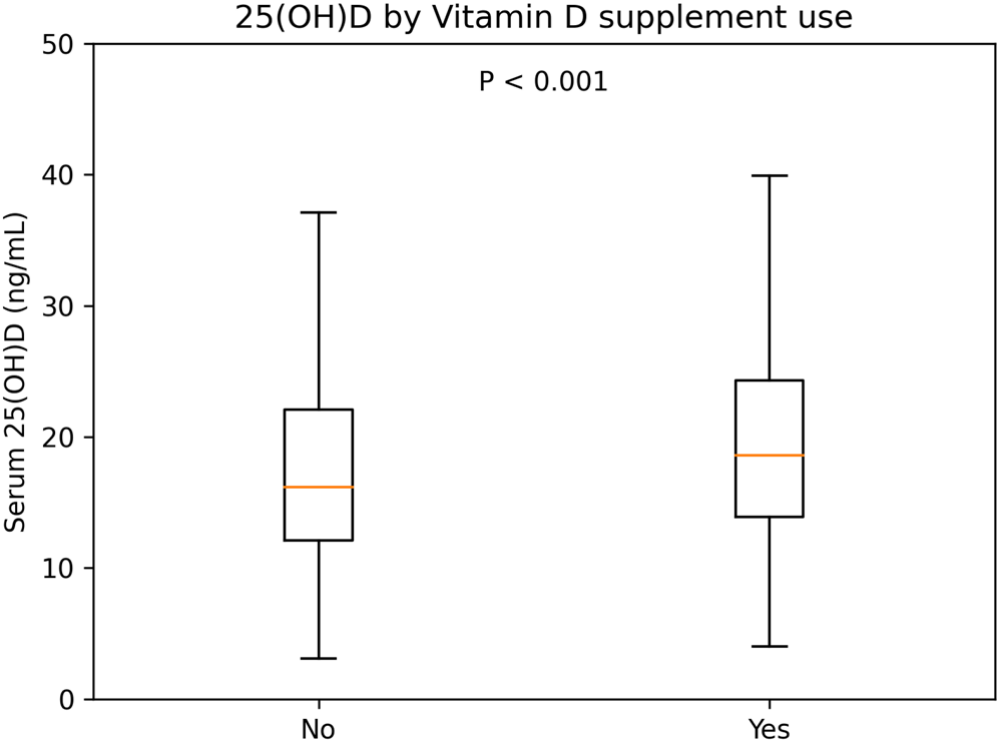
Seasonal distribution of vitamin D status in women attending infertility clinics in Japan. Vitamin D status was categorized into three mutually exclusive groups based on serum 25(OH)D concentration: Deficient (< 20 ng/mL), insufficient (20–< 30 ng/mL), and sufficient (≥ 30 ng/mL). Bars represent the proportion of women in each category by season (winter, spring, summer, autumn; defined as in Figure 1B). Differences in the 3‐category distribution across seasons were evaluated using the chi‐square test; *p* values are shown on the graph.

Among women with complete information on both vitamin D and multivitamin supplementation and a valid serum 25(OH)D measurement (*n* = 2841), 2701 (95.1%) reported no use of either vitamin D or multivitamin supplements, whereas 140 (4.9%) reported current use of at least one vitamin D–containing preparation (Table 3). In the supplement‐free group, the median 25(OH)D level was 15.3 ng/mL (IQR, 12.0–20.1), compared with 21.7 ng/mL (IQR, 14.6–27.9) among supplement users (Mann–Whitney *U* test, *p* < 0.001; Table 3, Figure 2).

**TABLE 3:** Vitamin D status according to vitamin D–containing supplement use (complete‐case subset).

	Non‐users	Users	*p*
*N*	2701	140	
Serum 25(OH)D, median (IQR), ng/mL	15.3 (12.0–20.1)	21.7 (14.6–27.9)	< 0.001
Vitamin D status, *n* (%)			< 0.001
Deficient (< 20)	2013 (74.5%)	62 (44.3%)	
Insufficient (20–< 30)	527 (19.5%)	50 (35.7%)	
Sufficient (≥ 30)	161 (6.0%)	28 (20.0%)	

When vitamin D status was presented as mutually exclusive categories (deficient < 20, insufficient 20 to < 30, sufficient ≥ 30 ng/mL), the distribution differed significantly between groups (chi‐square test, *p* < 0.001; Table 3). Specifically, vitamin D deficiency was observed in 74.5% (2013/2701) of non‐users versus 44.3% (62/140) of users, while sufficient levels (≥ 30 ng/mL) were observed in 6.0% (161/2701) of non‐users versus 20.0% (28/140) of users (Table 3). Notably, 94.0% (2540/2701) of women in the supplement‐free group were vitamin D insufficient (< 30 ng/mL). These findings indicate that the very high burden of low vitamin D status and the overall distribution of 25(OH)D in this infertility cohort are not driven by supplement users.

In the overall cohort, the distribution of serum 25(OH)D status categories (deficient/insufficient/sufficient) did not differ significantly by diminished ovarian reserve status (chi‐square test, *p* = 0.238; Table S1).

Within this complete‐case supplementation subset, supplement use did not differ by diminished ovarian reserve status (chi‐square test, *p* = 0.965), and BMI distributions were similar between supplement users and non‐users (Mann–Whitney *U* test, *p* = 0.544; Table S2).

### 3.5 Factors Associated With Vitamin D Deficiency

Multivariable logistic regression results are summarized in Table 4. In the primary model adjusting for age, BMI, infertility diagnosis categories, and clinic (complete‐case *n* = 12 289), higher BMI was independently associated with vitamin D deficiency (aOR 1.05 per 1 kg/m^2^ increase, 95% CI 1.04–1.06, *p* < 0.001), whereas older age was associated with lower odds of deficiency (aOR 0.98 per 1‐year increase, 95% CI 0.97–0.99, *p* < 0.001). Tubal factor infertility was inversely associated with deficiency (aOR 0.85, 95% CI 0.76–0.96, *p* = 0.006), and diminished ovarian reserve also showed an inverse association (aOR 0.86, 95% CI 0.75–0.97, *p* = 0.018).

**TABLE 4:** Factors associated with vitamin D deficiency (serum 25(OH)D < 20 ng/mL).

Variable	Model 1 aOR (95% CI)	*p*	Model 2 aOR (95% CI)	*p*
Age (per 1‐year increase)	0.98 (0.97–0.99)	< 0.001	0.98 (0.98–0.99)	0.001
BMI (per 1 kg/m^2^ increase)	1.05 (1.04–1.06)	< 0.001	1.05 (1.03–1.06)	< 0.001
Endometriosis	0.96 (0.84–1.11)	0.598	0.95 (0.81–1.11)	0.527
Polycystic ovary syndrome (PCOS)	0.97 (0.82–1.14)	0.724	0.95 (0.79–1.14)	0.576
Diminished ovarian reserve	0.86 (0.75–0.97)	0.018	0.91 (0.77–1.09)	0.321
Corpus luteum insufficiency	1.37 (0.73–2.56)	0.322	1.47 (0.52–4.15)	0.472
Ovulatory factor	0.95 (0.80–1.13)	0.558	0.86 (0.67–1.10)	0.227
Tubal factor	0.85 (0.76–0.96)	0.006	0.83 (0.73–0.94)	0.004
Uterine factor	0.94 (0.79–1.11)	0.456	0.88 (0.67–1.15)	0.345
Male factor	0.98 (0.90–1.07)	0.677	0.90 (0.82–0.99)	0.027
Spring vs. Winter			0.82 (0.65–1.03)	0.094
Summer vs. Winter			0.87 (0.68–1.10)	0.252
Autumn vs. Winter			0.59 (0.47–0.74)	< 0.001

*Note:* Model 1 adjusted for age, BMI, infertility diagnosis categories, and clinic (complete‐case *n* = 12 289). Model 2 additionally adjusted for season of measurement (complete‐case *n* = 8956). Adjusted odds ratios (aORs) for clinic indicators are not shown.

In the secondary model additionally adjusting for season (complete‐case *n* = 8956), autumn was associated with substantially lower odds of vitamin D deficiency compared with winter (aOR 0.59, 95% CI 0.47–0.74, *p* < 0.001), while the associations for BMI (aOR 1.05, 95% CI 1.03–1.06, *p* < 0.001) and age (aOR 0.98, 95% CI 0.98–0.99, *p* = 0.001) remained consistent. Tubal factor remained inversely associated (aOR 0.83, 95% CI 0.73–0.94, *p* = 0.004), and male factor showed a modest inverse association (aOR 0.90, 95% CI 0.82–0.99, *p* = 0.027; Table 4).

## 4 Discussion

This large multicenter cross‐sectional study provides robust real‐world evidence that low serum [25(OH)D] is highly prevalent among women attending infertility clinics in Japan. The overall burden of deficiency and insufficiency observed in our cohort, together with clear monthly/seasonal oscillations, underscores that vitamin D status in infertility practice is likely influenced by broad population‐level determinants and lifestyle environments affecting reproductive‐aged women.

### 4.1 Relationship to Prior Infertility‐ and ART‐Focused Evidence

Interest in vitamin D in reproductive medicine has been driven by the expression of vitamin D receptors and metabolizing enzymes in reproductive tissues and by proposed effects on folliculogenesis, steroidogenesis, endometrial receptivity, and immune modulation. Nevertheless, clinical evidence linking vitamin D status to infertility phenotypes and ART outcomes remains heterogeneous. Several observational studies and meta‐analyses in assisted reproduction suggest that higher 25(OH)D levels may be associated with improved clinical pregnancy and live birth rates, although effect sizes are modest and heterogeneity across cohorts, thresholds, and assay methods remains substantial [3, 4]. Notably, multiple meta‐analyses in ART populations have reported that higher serum 25(OH)D is associated with better pregnancy outcomes, including clinical pregnancy and/or live birth, while also emphasizing between‐study heterogeneity and differences in cutoffs and assays [11, 12, 13, 14].

Beyond pregnancy outcomes, one clinically relevant infertility‐oriented pathway is immune regulation at both systemic and endometrial levels. Ikemoto et al. reported that vitamin D insufficiency in infertile women was associated with an abnormally elevated Th1/Th2 cell ratio, and that continuous daily vitamin D supplementation achieving serum 25(OH)D > 30 ng/mL was accompanied by improvement of the Th1/Th2 balance in both peripheral blood and the local endometrium [15]. This finding is particularly relevant to infertility practice because it supports a biologically plausible link between vitamin D status and immune environments that may influence implantation and early reproductive processes, while also illustrating that supplementation can meaningfully shift immune profiles when sustained and when a target level is achieved.

Interventional evidence is also mixed: a recent meta‐analysis of vitamin D supplementation in infertile women reported potential improvements in clinical pregnancy in some subgroups while emphasizing variability in baseline status, dosing, and outcome definitions [16]. In PCOS, supplementation meta‐analyses suggest possible benefits on ovulation and pregnancy alongside endocrine improvements, yet the overall certainty of evidence is limited, and higher‐quality trials are still needed [5]. Taken together, the literature supports a plausible association between vitamin D status and reproductive outcomes in infertility/ART settings, but the magnitude and consistency of benefit—particularly for live birth—appear sensitive to baseline deficiency, achieved [25(OH)D] levels, and study design.

Importantly, our study population consists of women attending infertility clinics, and our findings are best interpreted within this context. While studies in recurrent pregnancy loss (RPL) have also linked low [25(OH)D] to immune dysregulation and early pregnancy maintenance, these populations differ clinically from infertility cohorts [17, 18, 19]. In our multicenter database, exploratory analyses of serum [25(OH)D] according to the number of previous miscarriages did not reveal a consistent, clinically meaningful gradient toward lower vitamin D levels in women with multiple losses, and miscarriage history was therefore not incorporated as a primary covariate in the multivariable models (data not shown).

Our study primarily characterizes the real‐world burden and determinants of low vitamin D status in infertility practice rather than testing an intervention effect on fertility outcomes. Nevertheless, the immune and endometrial pathways suggested in infertile women—such as Th1/Th2 imbalance that may be modifiable when sustained supplementation achieves > 30 ng/mL—provide a mechanistic rationale for future targeted trials, and our prevalence data offer essential context for designing such studies in clearly deficient infertility subgroups.

Thus, while our multicenter data highlight a large and clinically relevant burden of low 25(OH)D in infertility practice, they also reinforce the need to interpret a single 25(OH)D value cautiously and to avoid positioning it as a stand‐alone infertility biomarker.

### 4.2 Seasonal Pattern and Interpretation

We identified clear monthly/seasonal differences, with the highest median levels in early autumn and the lowest levels in winter. This pattern mirrors our prior single‐center report and is biologically consistent with seasonal variation in ambient UVB exposure [7, 9, 20]. From a clinical standpoint, these findings favor a nuanced interpretation of testing: vitamin D status assessed in winter may overestimate long‐term deficiency for some patients, whereas persistently low levels in late summer/autumn may more strongly indicate chronic insufficiency. This perspective supports clinically practical counseling that integrates diet, safe sunlight exposure, and individualized supplementation rather than reflexive labeling based on a single seasonal measurement.

### 4.3 Comparison With Previous Studies and Interpretation of Prevalence

The high prevalence of vitamin D deficiency in our cohort should be interpreted in the context of a global public health pattern affecting women of reproductive age rather than as an infertility‐specific phenotype alone. Large pooled analyses of population‐based studies confirm that vitamin D deficiency remains common worldwide, with substantial heterogeneity by latitude and season, and women are consistently identified as a vulnerable group [7]. In Western populations, recent syntheses estimate that the prevalence of 25(OH)D < 50 nmol/L (20 ng/mL) is approximately 24% in the United States, 37% in Canada, and 40% in Europe, underscoring that low vitamin D is not unique to Japan [20]. A European Calcified Tissue Society position statement similarly reports broad regional gradients, with lower prevalence in Northern Europe and substantially higher rates in parts of Southern/Eastern Europe and the Middle East [21]. Evidence from other Asian settings further supports the notion that younger women may be particularly prone to low vitamin D status; Korean national data have highlighted a high burden of insufficiency/deficiency among younger age groups, and Chinese studies of women of childbearing age report high rates with marked seasonal amplification [22, 23].

Within this international context, Japan appears to share—and in some respects intensify—these modern lifestyle‐driven risks. In healthy Japanese young women, seasonal assessments indicate strikingly high deficiency rates across the year, with particularly severe dips in winter, and frequent sunscreen use and sun‐avoidant behaviors likely contributing to persistently low 25(OH)D levels [24]. Studies in pregnant Japanese women also demonstrate a severe burden of deficiency and emphasize the influence of limited sunlight exposure and dietary patterns [25, 26]. General‐population data likewise support substantial seasonal oscillations in Japan [27, 28]. Compared with some Western settings, Japan has less widespread vitamin D food fortification, and this structural difference, combined with lifestyle patterns among young women, may further contribute to the high prevalence observed across clinics [29].

Taken together, these findings suggest that vitamin D deficiency among women attending infertility clinics may be better understood as part of a broader public health pattern rather than an infertility‐specific phenotype. This perspective is important because it reframes the clinical meaning of testing in infertility care. On one hand, the consistently high prevalence across populations strengthens the case for recognizing low vitamin D status as a common and potentially modifiable health issue in young women. On the other hand, the near‐ubiquitous nature of low [25(OH)D] raises a critical practical question: if most reproductive‐aged women are deficient by conventional thresholds, a single measurement may have limited discriminatory value for predicting short‐term fertility outcomes and may function more as a marker of general lifestyle and metabolic risk than as a direct infertility biomarker. This interpretation is consonant with recent guidance and consensus statements emphasizing uncertainty regarding optimal targets for many non‐skeletal outcomes and recommending a risk‐stratified approach rather than indiscriminate routine screening in otherwise healthy populations [1, 30]. Our previous single‐center infertility study similarly demonstrated robust seasonal variation without a straightforward association with ovarian reserve or implantation‐related immune markers, supporting a cautious interpretation of causality for reproductive endpoints [9].

### 4.4 Factors Associated With Vitamin D Deficiency

Our multivariable analyses identified higher BMI as a consistent factor associated with vitamin D deficiency, while age showed a modest inverse association. These findings align with previous population‐level evidence linking adiposity to lower 25(OH)D, reinforcing that vitamin D status in infertility practice should be interpreted within a broader metabolic and lifestyle context [8, 31]. In addition, in this large real‐world multicenter cohort, the proportion of women reporting any nutritional supplement use, including vitamin D preparations, increased across age categories, supporting the interpretation that older infertility patients may be more health‐conscious and more likely to receive or adopt nutritional counseling and supplementation. The markedly higher 25(OH)D levels and lower, though still substantial, prevalence of deficiency among supplement users in our cohort are consistent with this interpretation. This pattern is consistent with population‐based surveys in other settings, which have reported that dietary supplement use is particularly common among older women and tends to co‐occur with other health‐conscious behaviors and preventive health service use [32, 33, 34, 35]. Importantly, vitamin D–containing supplement users represented only a small fraction of women with complete supplementation data, and vitamin D deficiency remained common even among users. Therefore, the high overall burden of low 25(OH)D in this cohort is unlikely to be an artifact driven by supplementation patterns. With respect to infertility diagnosis categories, the modest inverse associations observed for tubal factor, male factor, and diminished ovarian reserve (DOR) should be interpreted with particular caution. For DOR, this finding appears counterintuitive given prior reports linking lower vitamin D status with impaired ovarian reserve and follicular function [36, 37]. However, it is plausible that women labeled with DOR in routine practice were more frequently targeted for lifestyle advice or supplementation, partially mitigating deficiency risk. In couples with tubal or male factor infertility, a clearly identifiable non–vitamin D etiology may also dominate the clinical picture, such that subtle differences in female vitamin D status are less tightly coupled to infertility risk and may instead reflect residual confounding by BMI, age, or unmeasured lifestyle factors. In addition, infertility diagnosis categories were recorded in routine practice and may vary in definition and completeness across clinics; thus, these associations should be viewed primarily as hypothesis‐generating signals rather than evidence of etiologic or protective effects. Overall, these diagnosis‐specific associations are best viewed as hypothesis‐generating rather than evidence of a causal protective effect of particular infertility categories on vitamin D status.

### 4.5 Clinical Implications

From a pragmatic standpoint, our findings suggest that the value of measuring 25(OH)D in infertility clinics may lie less in short‐term prediction of ART outcomes and more in risk‐stratified health counseling for reproductive‐aged women. Contemporary endocrine guidance and international consensus statements emphasize uncertainty regarding optimal 25(OH)D targets for many non‐skeletal outcomes and caution against indiscriminate population‐wide screening, while supporting selective assessment for higher‐risk groups [1, 30]. In this context, the near‐ubiquitous deficiency observed in our cohort argues for a shift from “test‐and‐label” toward “test‐and‐contextualize,” where results are interpreted alongside BMI, season of measurement, lifestyle patterns, and dietary adequacy.

This approach can be operationalized using a simple interpretive framework. First, clinicians should recognize strong seasonal fluctuation and avoid over‐interpreting a single winter low value as evidence of a stable, infertility‐specific abnormality. Second, persistently low values in late summer to autumn may more strongly suggest chronic insufficiency and may justify structured counseling on diet, safe sunlight exposure, and supplementation. Third, because higher BMI is consistently associated with lower 25(OH)D across populations, weight‐related counseling should be integrated into vitamin D conversations in infertility care.

Regarding therapeutic implications, existing evidence suggests that vitamin D supplementation may improve clinical pregnancy in some infertile populations [16] and may be more notably beneficial in PCOS, though trial quality, baseline status, and dosing regimens vary substantially across studies [5, 16]. In addition, immune‐focused data in infertile women indicate that sustained daily supplementation achieving serum 25(OH)D > 30 ng/mL may improve Th1/Th2 balance in both peripheral blood and the endometrium, providing a mechanistic rationale for considering supplementation in clearly deficient patients [15]. Thus, our data support a measured, phenotype‐aware approach: supplementation might be most defensible for women with clear deficiency plus additional risk factors (e.g., higher BMI, limited sunlight exposure, restricted diet, PCOS), rather than as a universal strategy intended to enhance ART success in all comers.

### 4.6 Strengths and Limitations

This study has several strengths. First, the large multicenter sample size provides one of the most robust real‐world estimates of vitamin D deficiency in women attending infertility clinics in Japan. Second, the inclusion of month/season of measurement allows clinically relevant interpretation of temporal variability in 25(OH)D distributions. Third, the use of a standardized data sheet across clinics improves comparability of infertility‐related diagnoses and key covariates.

Several limitations should also be acknowledged. The retrospective, cross‐sectional nature of the database precludes causal inference and limits our ability to determine whether low vitamin D directly contributes to infertility phenotypes or affects ART outcomes. Missing data were unavoidable in this real‐world setting, and complete‐case multivariable analyses may have introduced selection bias. In addition, we also lacked granular information on sun exposure habits, detailed dietary intake, and the dose or duration of vitamin D supplementation, which are known drivers of vitamin D status. Inter‐clinic differences in assay methods or testing indications could also have contributed to residual heterogeneity. Finally, because vitamin D deficiency is highly prevalent in young Japanese women even outside infertility settings, our findings should not be interpreted as evidence of an infertility‐specific vitamin D phenotype.

### 4.7 Future Directions

Future work should focus on clarifying when and for whom vitamin D assessment meaningfully changes clinical decisions in reproductive care. Prospective cohort studies linking baseline [25(OH)D] with ART and natural conception outcomes—while controlling for seasonal timing, BMI, sun exposure, and dietary patterns—would help determine the incremental predictive value of vitamin D beyond lifestyle and metabolic risk markers.

Randomized trials in clearly defined high‐risk subgroups (e.g., women with severe deficiency, higher BMI, or PCOS) are needed to identify optimal dosing strategies and to confirm whether supplementation improves reproductive endpoints or primarily benefits broader cardiometabolic and skeletal health. Given that Japan has less widespread vitamin D food fortification compared with some Western settings, policy‐relevant research evaluating feasible fortification or structured preconception nutritional programs may also be warranted. Finally, integrating vitamin D assessment into a broader, multi‐nutrient preconception framework—alongside folate/one‐carbon metabolism, iron status, and lifestyle risk profiling—may better reflect the real‐world determinants of reproductive health than a single‐nutrient, single‐threshold model.

Taken together, this large multicenter real‐world dataset demonstrates that low serum [25(OH)D] is highly prevalent among women attending infertility clinics in Japan and shows clear monthly/seasonal variation, with adiposity‐related vulnerability as a consistent correlate. These findings suggest that vitamin D deficiency in infertility practice is best understood as a common and potentially modifiable health issue in reproductive‐aged women rather than a condition‐specific infertility biomarker. Clinically, a single [25(OH)D] measurement should therefore be interpreted with explicit attention to season and BMI and used primarily to guide pragmatic, risk‐stratified counseling on diet, safe sunlight exposure, weight‐related risk, and supplementation when appropriate, rather than as a stand‐alone predictor of short‐term fertility or ART outcomes. Prospective studies and targeted trials in clearly defined high‐risk subgroups are warranted to determine when vitamin D assessment and intervention meaningfully improve reproductive endpoints and broader health.

## Conflicts of Interest

The authors declare no conflicts of interest.

## Supporting information


**Table S1:** Association between serum 25(OH)D status and diminished ovarian reserve (DOR).

**Table S2:** Association of vitamin D–containing supplement use with diminished ovarian reserve (DOR) and body mass index (BMI) in the complete‐case supplementation subset.

## Data Availability

The data that support the findings of this study are available from the corresponding author upon reasonable request.
