# Supplementary material for: Prevalence and Determinants of Low Serum Vitamin D Among Women Attending Infertility Clinics in Japan: A Real‐World Multicenter Cross‐Sectional Study
Source: Reprod Med Biol. 2026 Feb 22;25(1):e70032. doi: 10.1002/rmb2.70032 (PMC12928063; doi:10.1002/rmb2.70032)
Supplement: Supplementary file 1 — Table S1: Association between serum 25(OH)D status and diminished ovarian reserve (DOR). [file RMB2-25-e70032-s002.docx]

**Supplementary Table S1. Association between serum 25(OH)D status and diminished ovarian reserve (DOR)**

Vitamin D status categories were defined as deficient (<20 ng/mL), insufficient (20–<30 ng/mL), and sufficient (≥30 ng/mL). Values are n (% within each vitamin D status category).

| **Serum 25(OH)D status** | **DOR (–)** | **DOR (+)** | **Total** | **P value** |
| --- | --- | --- | --- | --- |
| Deficient (<20) | 9,815 (85.2%) | 1,709 (14.8%) | 11,524 |  |
| Insufficient (20–<30) | 3,262 (85.0%) | 574 (15.0%) | 3,836 |  |
| Sufficient (≥30) | 1,646 (86.6%) | 255 (13.4%) | 1,901 |  |
| Total | 14,723 | 2,538 | 17,261 | 0.238^†^ |

^†^ P value calculated using the chi-square test.
