# Supplementary material for: Prevalence and Determinants of Low Serum Vitamin D Among Women Attending Infertility Clinics in Japan: A Real‐World Multicenter Cross‐Sectional Study
Source: Reprod Med Biol. 2026 Feb 22;25(1):e70032. doi: 10.1002/rmb2.70032 (PMC12928063; doi:10.1002/rmb2.70032)
Supplement: Supplementary file 2 — Table S2: Association of vitamin D–containing supplement use with diminished ovarian reserve (DOR) and body mass index (BMI) in the complete‐case supplementation subset. [file RMB2-25-e70032-s001.docx]

**Supplementary Table S2. Association of vitamin D–containing supplement use with diminished ovarian reserve (DOR) and body mass index (BMI) in the complete-case supplementation subset**

Complete-case subset included women with valid serum 25(OH)D measurement and complete information on vitamin D supplement and multivitamin use (n=2,841). Supplement users were defined as those reporting current intake of either a vitamin D supplement or a multivitamin containing vitamin D; non-users reported neither.

**A) Supplement use by diminished ovarian reserve (DOR)**

| **DOR** | **Non-users, n (%)** | **Users, n (%)** | **P value** |
| --- | --- | --- | --- |
| No | 1,867 (95.1%) | 96 (4.9%) |  |
| Yes | 834 (95.0%) | 44 (5.0%) |  |
| P value |  |  | 0.965^†^ |

^†^ P value calculated using the chi-square test.

**B) BMI by supplement use**

| **Variable** | **Non-users** | **Users** | **P value** |
| --- | --- | --- | --- |
| BMI, kg/m² | 21.0 (19.5–23.2) | 21.1 (19.5–22.9) | 0.544^‡^ |

Values are median (IQR) unless otherwise indicated.

^‡^ P value calculated using the Mann–Whitney U test.
